# Cognitive component of auditory attention to natural speech events

**DOI:** 10.3389/fnhum.2024.1460139

**Published:** 2025-01-06

**Authors:** Nhan Duc Thanh Nguyen, Kaare Mikkelsen, Preben Kidmose

**Affiliations:** Center for Ear-EEG, Department of Electrical and Computer Engineering, Aarhus University, Aarhus, Denmark

**Keywords:** auditory attention decoding, ERPs, ear-EEG, cognitive processing, P300

## Abstract

The recent progress in auditory attention decoding (AAD) methods is based on algorithms that find a relation between the audio envelope and the neurophysiological response. The most popular approach is based on the reconstruction of the audio envelope from electroencephalogram (EEG) signals. These methods are primarily based on the exogenous response driven by the physical characteristics of the stimuli. In this study, we specifically investigate higher-level cognitive responses influenced by auditory attention to natural speech events. We designed a series of four experimental paradigms with increasing levels of realism: a word category oddball paradigm, a word category oddball paradigm with competing speakers, and competing speech streams with and without specific targets. We recorded EEG data using 32 scalp electrodes, as well as 12 in-ear electrodes (ear-EEG) from 24 participants. By using natural speech events and cognitive tasks, a cognitive event-related potential (ERP) component, which we believe is related to the well-known P3b component, was observed at parietal electrode sites with a latency of ~625 ms. Importantly, the component decreases in strength but is still significantly observable in increasingly realistic paradigms of multi-talker environments. We also show that the component can be observed in the in-ear EEG signals by using spatial filtering. We believe that the P3b-like cognitive component modulated by auditory attention can contribute to improving auditory attention decoding from electrophysiological recordings.

## 1 Introduction

A remarkable aspect of human perception is our ability to segregate concurrent auditory objects and selectively attend to these objects. It is an ability we are readily familiar with and utilize in many situations, for example in “cocktail party” scenarios, to volitionally focus on the speaker(s) of interest. However, for individuals with hearing impairment, the ability to segregate sounds is often significantly reduced, even when using a hearing device to compensate for the elevated hearing threshold. This motivates the development of smarter hearing aids with integrated modules to decode the auditory attention of users. In turn, this increases interest in gaining a deeper understanding of how the processing of auditory stimuli impacts EEG recordings. The process of decoding auditory attention, based on electrophysiological signals, normally electroencephalography (EEG), is referred to as auditory attention decoding (AAD).

Previous work on AAD has successfully investigated the correlation between the recorded EEG and the envelope of the attended sound stream (Aiken and Picton, 2008; Ding and Simon, 2012) to identify the attended sound stream. However, this approach largely relies on how attention affects the early response to the physical attributes of the stimuli, rather than the subsequent cognitive processing related to the semantics of the speech of the audio. In this study, we have investigated the extent to which this cognitive processing can be estimated from real speech streams, to pave the way for future AAD implementations, in combination with the envelope following response.

As previously noted, the majority of state-of-the-art AAD methods rely on the increased entrainment to the envelope of attended sounds compared to unattended sounds (Ding and Simon, 2012). The impact of attention on the envelope-following response forms the foundation for research in the field of AAD. In AAD, the attended speech stream is determined as the one whose envelope has the highest correlation with the reconstructed envelope. The majority of studies have trained linear (O'Sullivan et al., 2015; Biesmans et al., 2017; Geirnaert et al., 2021a; Aroudi et al., 2019; O'Sullivan et al., 2017) and non-linear (de Taillez et al., 2020; Nogueira et al., 2019; Xu et al., 2022) models to predict the attended audio envelope from multi-channel EEG (called backward modeling). Other studies use linear models to predict the EEG based on the attended audio envelopes (called forward modeling) (Wong et al., 2018; Alickovic et al., 2019). Another approach, called canonical correlation analysis (CCA) (Hotelling, 1936), combines a backward model on the EEG and a forward model on the speech envelope to maximize the correlation of their outputs jointly (de Cheveigné et al., 2018). The CCA method has so far been the best method with a decoding accuracy of up to ~90% for a decision window length of 30 s tested on specific datasets in a well-controlled environment, with two competing speakers (Geirnaert et al., 2021b).

Since our investigation concerns the cognitive processing of the stimuli, our analysis relies heavily on Event-Related Potentials (ERPs), caused by the processing of specific auditory events. Due to the high temporal resolution (Woodman, 2010), there has been an interest in analyzing ERPs for investigating selective attention mechanisms. Extensive research has been dedicated to understanding which components and cognitive systems are influenced by attention. Some researchers (Broadbent, 1958; Treisman, 1964; Treisman and Geffen, 1967) have hypothesized an early selection mechanism when the sensory systems are overloaded by multiple inputs, leading to the selection of input for further processing. A study (Hillyard et al., 1973) found that the N1 component (occurring around 150 ms) was affected by the attention (amplitude was larger when the stimuli presented in the attended ear than those in the unattended ear). This supports a hypothesis of early selection for attention. However, another line of research has focused on attention mechanisms in the post-perceptual processes (Moray, 1959; Deutsch and Deutsch, 1963). Others (Luck and Kappenman, 2011) argued that selective attention could happen in any system in the brain when that system becomes overloaded. That means attention may happen at either the early or late stage or even both, depending on the nature of stimuli and task. For instance, in tasks combining several complicated cognitive tasks and responses, we might expect an overload in the memory and response systems and in consequence an impact on the late post-perceptual components. Therefore, late cognitive components like the P300 become relevant in the study of attention. The P300 component, also known as P3, is an endogenous potential, as it is related to cognitive processing rather than the physical attributes of a stimulus. More specifically, the P300 is thought to reflect processes involved in stimulus evaluation or categorization (Polich, 2007; Kutas et al., 1977). The P300 has a positive-going amplitude peaking around 300 ms, and the peak will vary in latency from 250 to 500 ms or more, depending on stimuli, task, and subject. Numerous studies have investigated how auditory attention affects P3. For instance, the more attention or resources allocated to process a stimulus, the larger the P3 amplitude is (Isreal et al., 1980; Kramer et al., 1983; Mangun and Hillyard, 1990). Following the early findings, recent studies have investigated the manipulation of attention to different ERP components to see the differences between different neurological population groups. Schierholz et al. (2021) has demonstrated the effect of attention on N1, N2, P2, and P3 ERP components on both cochlear-implant (CI) users and normal-hearing (NH) people. The study has also found that there is enhanced attentional modulation on N1 latency in CI users compared to the NH group. Another study (Vanbilsen et al., 2023) has investigated the auditory attention of neurological populations and found impairments in auditory processing in terms of magnitude and delay of the P3 component compared to the healthy group. This suggests that the P3 component could potentially be a reliable feature for AAD.

Currently, the majority of AAD research relies on traditional scalp EEG, which is obtrusive and uncomfortable for long recordings in real-world environments. This necessitates the development of less invasive methods for capturing EEG signals. As a response to this challenge, there has been an increasing interest in the development of miniaturized and wearable EEG devices that provide a discrete, unobtrusive, and user-friendly recording solution. One such solution is so-called ear-EEG systems Kappel et al. (2019), where EEG is recorded from electrodes placed in the ears. Numerous studies have compared ear-EEG and conventional scalp EEG, e.g. in terms of auditory middle and late latency ERPs, signal-to-noise ratio, power spectrum Mikkelsen et al. (2015) and P3 response Farooq et al. (2015). Additionally, in the field of AAD, in-ear single sensor Fiedler et al. (2017) and around-the-ear sensors Holtze et al. (2022) have successfully been demonstrated to unobtrusively monitor auditory attention to tone streams and continuous speech streams.

Inspired by the above-mentioned works on ERP methods for AAD, our study uses speech streams with different tasks and levels of realism, to methodically probe to which extent the cognitive processing of events (words) can be detected as an ERP. We consider the feasibility of this method not only in terms of how realistic the listening task can be but also in how “realistic” the recording setup can be made. This means both investigating a series of progressively more demanding and realistic listening scenarios, but also performing this investigation using both conventional scalp EEG and a dry-contact ear-EEG setup (Kappel et al., 2019), which is likely to be more relevant for future AAD implementations in, for instance, brain-controlled hearing aids.

## 2 Methods

### 2.1 Participants

Twenty-four native Danish-speaking subjects (26.87 ± 8.44 years, 13 male, five left-handed) participated in the study. All subjects provided written informed consent and reported normal ability and no neural disorders. The experimental protocol was approved by the Research Ethics Committee (Institutional Review Board) at Aarhus University, approval number 2021-79.

### 2.2 Experimental paradigms

The study comprised four experimental paradigms. The paradigms represent an increasing level of complexity, and all participants conducted all four experiments sequentially from paradigm 1 to 4.

#### 2.2.1 Paradigm 1: word category oddball

This paradigm was designed to be similar to the conventional oddball paradigm, in that subjects were presented with a sequence of two different classes of spoken words: *animal names* and *cardinal numbers*, or *color names* and *cardinal numbers* from a loudspeaker situated one meter in front of the subject. The animal names and color names were predefined as the *target* events, while the cardinal numbers were the *non-target* events. The target and non-target events in this context play similar roles as oddball and standard events in the classical oddball paradigm. However, unlike conventional oddball paradigms, the discrimination between the target and non-target events was not based on the physical attributes of the stimuli but rather on the semantics of the stimuli. The stimuli for each trial were generated by randomly mixing twenty target and non-target events, with the number of target events between 2 and 5 and the first two always being non-targets. The proportion of target events was chosen based on two criteria: (1) to provide enough data for analysis, and (2) not too many since less probable events would produce a larger cognitive response. The distance between two consecutive events was random and uniformly distributed between 0.8 and 1.2 s while keeping the total length of the trial at 20 s. In each trial, the subject was asked to pay attention to the target events and passively count them. At the end of each trial, the subject reported the number of target events and received feedback on their accuracy. The counting task and feedback were used to encourage the subject to remain engaged in the task. There were sixteen trials in this paradigm. The target events in the first eight trials were animal names and the target events in the last eight trials were color names.

#### 2.2.2 Paradigm 2: word category with competing speakers

Paradigm 2 was an extension of Paradigm 1, using similar sequences of twenty discrete spoken words. However, instead of a single stream of words, two competing streams were presented simultaneously by two speakers located at equal distances on either side of the subject, placed 60 degrees to the left and right. In each trial, the subject was asked to pay attention to only the target events in one of the streams and disregard the other stream. The subject was instructed to passively count the number of target events in the attended stream and report the count after the trial. The target events in the twenty trials were balanced between the two classes of events. Additionally, the instruction on which stream to attend was randomized but balanced between the left and right speakers to avoid bias toward a particular listening direction.

#### 2.2.3 Paradigm 3: competing speech streams with targets

In Paradigm 3, the setup was similar to the setup of Paradigm 2. The subject was presented with two competing streams from the same two speakers as in Paradigm 2. However, in this case, the stimuli in each speaker were not sequences of spoken words but snippets of different stories and each snippet had a duration of ~20 s. Each trial had one class of words predefined as target words. For instance, a target class could be *human names*. In each trial, the subject was asked to attend to one of the two streams (left or right) and focus on the target words of that stream. At the end of the trial, the subject answered a question about the target words and received feedback. There were four classes of target words: animal names, human names, color names, and plant species, distributed over five different stories. The story of the attended stream in each trial continued from where it ended in the previous trial. This made the stream easier to follow and attend. There were 20 snippets in total. Each snippet appeared twice in two different trials: one time as the attended stream and one time as the unattended stream. The attended stream was also randomized and balanced between the left and right speakers.

#### 2.2.4 Paradigm 4: competing speech streams without targets

Paradigm 4 was designed to simulate a real-world scenario of selective listening in a setting with multiple sound sources. Two competing streams from the same loudspeaker setup as utilized in Paradigm 2 and 3 were presented to the subject, each stream contained a single-speaker narrative. No particular target words were specified for this paradigm. The subject was instructed to attend to one stream while disregarding the other in each trial. Following each trial, the subject was probed with a question about the content and was provided with feedback. The data collected from this paradigm was not employed in any analyses or results presented in this paper but is merely described here for completeness.

The motivation for this experimental design was to investigate the cognitive processing of speech events. Advancing from Paradigm 1 to 3, the experimental complexity was increased in terms of task difficulty and how realistic it was compared to a real-world scenario, thus enabling investigation of the impact of the attention mechanism on the strength and distinctiveness of the cognitive component.

### 2.3 Stimuli

The stimuli used in the whole experiment were in Danish and synthesized using the Google Text-to-Speech tool v2.11.1 (Text-to-Speech AI, 2022). The voice configuration was randomly selected between *da-DK-Wavenet-A* (female) and *da-DK-Wavenet-C* (male) to generate each snippet in Paradigm 3 and 4. In the end, there were 14 out of 20 male voice snippets in Paradigm 3 and 24 out of 40 male voice snippets in Paradigm 4. Each voice configuration was selected to generate every single word in Paradigm 1 and 2 once. The speed was set at 0.85 and the authenticity was verified by native Danish speakers to make sure that the pronunciation was natural and clear. In this process, minor changes were made to the text to increase speech quality. The target words in the paradigms were selected to be short (1 or 2 syllables) to resemble the oddball events. The details of stimuli generation are outlined below:

- Paradigms 1 and 2 shared the same words. The waveform of each word was normalized to have the same root mean square (RMS) amplitude. The normalized sound files were then concatenated to fit the experimental design.
- The stimuli for Paradigms 3 and 4 were generated similarly. Text scripts of the stories were first made and then used for audio synthesis using the text-to-speech tool. The audio files were then sliced into different snippets and normalized to have the same RMS amplitude. The text scripts for Paradigm 3 were created by native Danish speakers. For Paradigm 4, the text scripts were sourced from two books (The Hobbit and Northern Lights) and excerpts from Danish radio broadcast news covering politics, society, education, sports, and social networks. The word onset times, to be used in the subsequent ERP analysis, was extracted from the text-to-speech tool.

**Figure 1:**
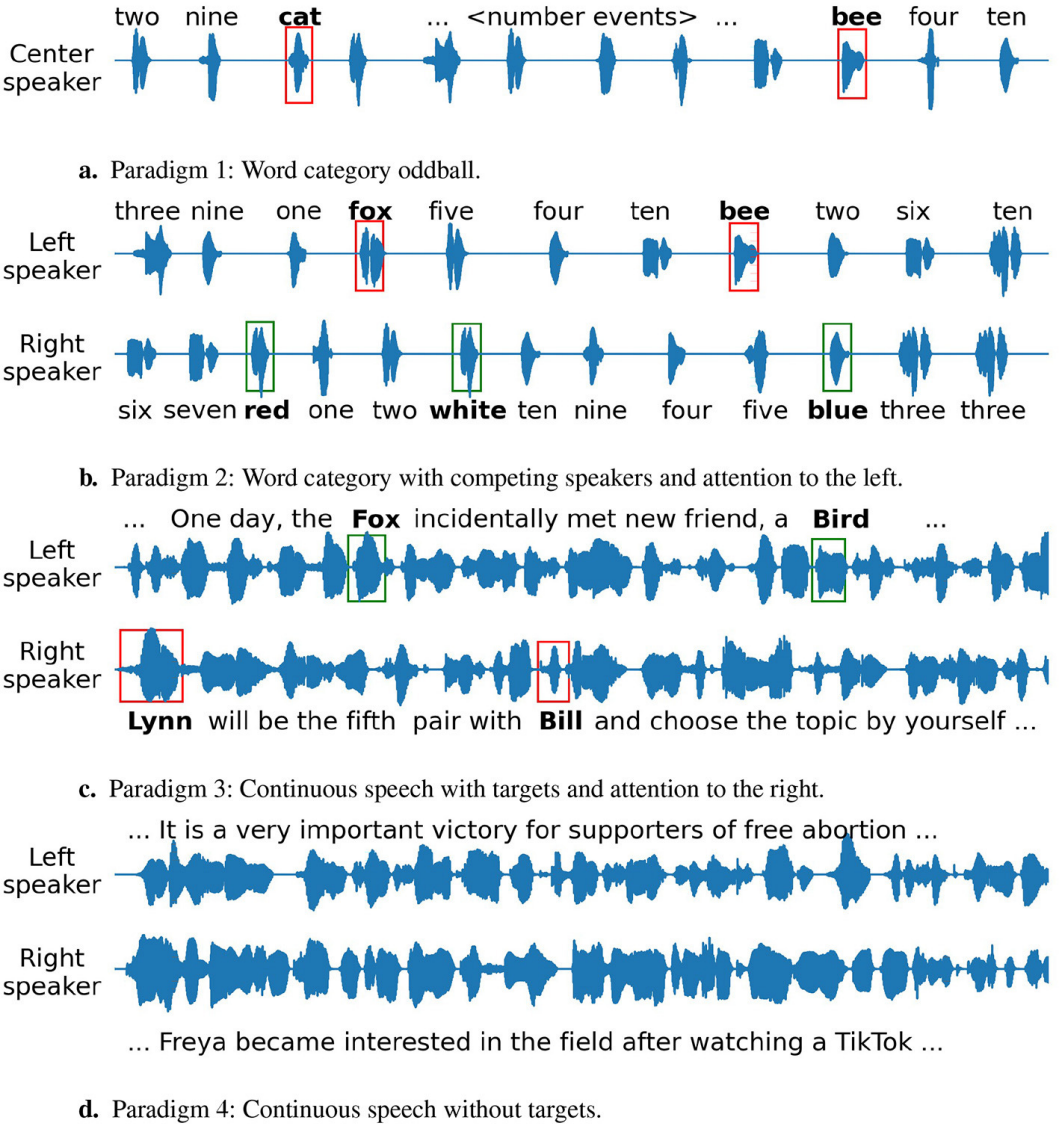
Illustration of the stimuli for the four paradigms. The text above or below the waveforms shows the corresponding word utterance. The red and green boxes indicate attended and unattended target words respectively. **(A)** Paradigm 1: Word category oddball. **(B)** Paradigm 2: Word category with competing speakers and attention to the left. **(C)** Paradigm 3: Continuous speech with targets and attention to the right. **(D)** Paradigm 4: Continuous speech without targets.

An illustration of the stimuli for all paradigms is shown in Figure 1.

### 2.4 Experimental setup and data acquisition

**Figure 2:**
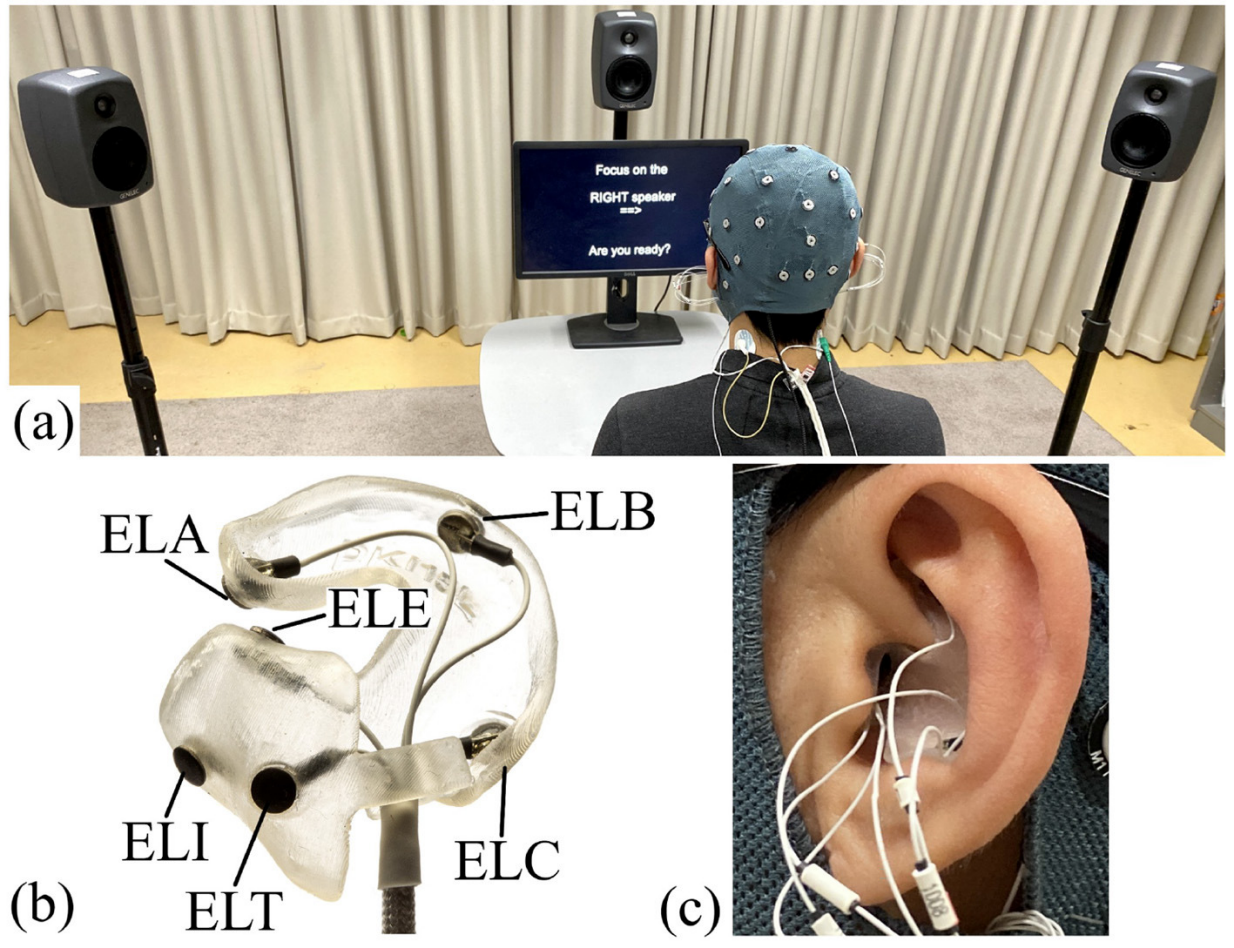
Experimental setup: **(A)** Overall setup. **(B)** Example of an earpiece for the left ear with electrodes in positions A, B, C, T, E, and I (according to the electrode position system proposed in Kidmose et al. (2013)). **(C)** Example of an earpiece mounted in the ear.

Participation in the study comprised two visits to the lab. At the first visit, an ear impression was taken to create individualized earpieces, which were used as part of the ear-EEG device (Kappel et al., 2019) to record EEG signals from the ears. The EEG was recorded at the second visit. The recording room was an acoustically shielded listening room with 0.4 s of reverberation time. The subject was seated in a chair in front of the loudspeakers, see Figure 2A. The EEG was recorded concurrently from 32 scalp electrodes and a left and right ear-EEG earpiece with six electrodes on each earpiece, see Figures 2B, C. EEG data were collected using two TMSi Mobita amplifiers. Because the scalp and ear-EEG electrodes were made of different materials (*Ag*/*AgCl* and *IrO*_2_, respectively), they were connected to separate amplifiers. However, the two amplifiers shared a common electrode position that had a combined *Ag*/*AgCl* and *IrO*_2_ electrode, which was placed at the Fpz location. This allowed for the scalp and ear-EEG data to be combined during post-processing. The 32 scalp electrodes were located according to the 10/20 system. Figure 2 shows the details of the experimental setup. The experiments were implemented in Python using the PsychoPy open-source platform v2022.1.4 (Peirce et al., 2019).

All 24 subjects completed all four experiments. The EEG sampling rate was 1,000 Hz for both amplifiers and referenced to the average channels within each amplifier as the default setup of the amplifiers. The data were then off-line re-referenced to the common reference electrode in each amplifier, cut into trial blocks, and concatenated to discard the data between trials. Data from each of the four paradigms were saved in separate files following the BIDS format (Gorgolewski et al., 2016).

### 2.5 Data analysis

The percentage of correct answers from each participant was used as a *post hoc* data exclusion criterion. In the following, we outline the analysis pipeline used in the data analyses:

1. Reference scalp EEG data to the averaged channel of all scalp channels and ear-EEG data to the averaged channel within each ear.
2. Apply zero-phase FIR bandpass filter with passband corner frequencies 0.1 and 40 Hz.
3. Apply the Independent Component Analysis (ICA) method to remove eye components (only for scalp EEG)
4. Epoch data from –200 ms to 1 second around the event onset and group into the two experimental conditions.
5. Apply baseline correction 200 ms pre-stimulus.
6. Apply peak-to-peak epoch rejection. Epochs containing min-max amplitude difference values above 200 μV were rejected across all channels.
7. Calculate the individual average ERPs and grand average ERPs for each group by averaging across epochs within the group.

The processing pipeline was applied to the saved dataset from the data acquisition step. Note that the referencing applied before saving the data in BIDS format does not affect the subsequent analysis in this manuscript, as the scalp EEG and ear EEG in step (1) are re-referenced to the average of the scalp and ear, respectively. Thereby the scalp- and ear-EEG signals become independent.. In step (3), the ICA method was performed on the scalp EEG data using the *fastICA* method (Hyvarinen, 1999). ICs with a Pearson correlation coefficient with the electrooculography (EOG) channel larger than 0.8 were considered as EOG artifacts. The measurement space signals were reconstructed, using the mixing matrix, by leaving out the IC's identified as EOG artifacts. The processing pipeline was implemented using Python 3.9.9 and the supported library MNE-Python v1.2.0 (Gramfort, 2013). In step (4), the event onset was determined as the onset of the sound of the corresponding word in the speech by using the text-to-speech tool during the stimuli synthesis. The baseline correction in step (5) was applied by subtracting the mean of the baseline period from the entire epoch.

### 2.6 Electrode configuration selection

#### 2.6.1 Scalp EEG

The initial analysis of scalp EEG was based on data recorded from the Pz electrode referenced to the average of the scalp electrodes. The choice of electrode configuration is justified by the design of the experimental paradigms resembling the structure of conventional P3b paradigms, from which it is well known that the peak of the P3b wave is largest in the central parietal region (Polich, 2007).

#### 2.6.2 Ear-EEG

In general, EEG signals and ERPs estimated from ear-EEG recordings have smaller amplitudes and lower signal-to-noise ratios as compared to scalp EEG. This is mainly due to small electrode distances, lower spatial coverage of the scalp, and higher electrode impedances (Kappel et al., 2019). Additionally, due to inter-subject variability in the ear anatomy, there is a certain variation in the placement of the ear electrodes, which adds to the inter-subject variability of the ear-EEG signals. To optimize the SNR of the ERP waveform, we applied a spatial filter (Biesmans et al., 2015). The spatial filter was optimized for each ear individually, and computed in the following way:

Let *C* be the number of channels, *N* be the number of samples within the duration of an epoch, $E_{\text{T}}$ and $E_{\text{N}}$ be the sets of the *target* and *non-target* epoch indexes, *n*_T_ and *n*_N_ be the number of the *target* and *non-target* epochs, ${\text{X}}_{i}\in {\mathbb{R}}^{C\times N}$ be an epoch of multi-channels ear-EEG signals and **w**∈ℝ^*C*×1^ be the spatial filter (weighting vector). The goal of the spatial filtering method is to find the optimal **w** to maximize the ratio of output energy of the target ERP to the output of non-target ERP and other neural activities:


(1) $$\hat{w}=\;\underset{w}{\text{argmax}}\frac{{\left\|X_{\text{T}}^{⊺}w\right\|}_{2}^{2}}{\frac{1}{n_{\text{N}}}\sum_{i\in {ℰ}_{\text{N}}}{\left\|X_{i}^{⊺}w\right\|}_{2}^{2}},$$

with ${\text{X}}_{\text{T}}=\frac{1}{n_{\text{T}}}\underset{i\in E_{\text{T}}}{\sum }{\text{X}}_{i},{\text{X}}_{\text{T}}\in {\mathbb{R}}^{C\times N}$ the average target epoch. Equation 1 can be rewritten as:


(2) $$\hat{w}=\underset{w}{\text{argmax}}\frac{w^{⊺}X_{\text{T}}X_{\text{T}}^{⊺}w}{\frac{1}{n_{\text{N}}}\sum_{i\in {ℰ}_{\text{N}}}w^{⊺}X_{i}X_{i}^{⊺}w}$$

(3) $$=\underset{w}{\text{argmax}}\left(n_{\text{N}}\cdot \frac{w^{⊺}X_{\text{T}}X_{\text{T}}^{⊺}w}{w^{⊺}X_{\text{N}}X_{\text{N}}^{⊺}w}\right)$$

(4) $$=\underset{w}{\text{argmax}}\left(n_{\text{N}}\cdot \frac{w^{⊺}R_{\text{T}}w}{Nn_{\text{N}}w^{⊺}R_{\text{N}}w}\right),$$

with ${\text{X}}_{\text{N}}\in {\mathbb{R}}^{C\times Nn_{\text{N}}}$ the concatenation of non-target epochs along the time axes, ${\text{R}}_{\text{T}}\in {\mathbb{R}}^{C\times C}$ the auto-correlation matrix of averaged target epoch **X**_T_ and ${\text{R}}_{\text{N}}\in {\mathbb{R}}^{C\times C}$ the auto-correlation matrix of **X**_N_. From (4), it appears that if $\hat{\text{w}}$ is a solution, then any scalar multiplied with $\hat{\text{w}}$ is also a solution. The unique solution can be obtained by introducing the constraint ${\text{w}}^{⊺}{\text{R}}_{\text{N}}\text{w}=1$ (i.e., normalizing the output power of the non-target ERPs). Thus, the optimal spatial filter can be found by solving the following optimization problem:


(5) $$\hat{w}=\;\underset{w}{\text{argmax }}w^{⊺}R_{\text{T}}w,$$

subject to the constraint


(6) $$w^{⊺}R_{\text{N}}w=1.$$

Solving the optimization problem described by Equations 5, 6 using the Lagrange multipliers method leads to a generalized eigenvalue problem with the solution being the eigenvector which corresponds to the largest generalized eigenvalue of **R**_T_ and **R**_N_.

As the spatial filter is optimized based on data, a rigorous cross-validation technique is necessary to avoid overfitting. Therefore, the ERP was estimated from the ear-EEG using the following cross-task validation scheme:

1. For each paradigm and for each ear (left and right), a spatial filter was estimated based on data from the other two paradigms. Each filter was trained using six channels within an ear referenced to the average channel of that ear.
2. The spatial filters from step 1 were applied to all epochs from the paradigm that was not used for training those filters.
3. The individual ERPs and the grand average ERPs were calculated from the output of step 2 for each paradigm.

### 2.7 Statistical analysis

Cluster permutation test is a non-parametric statistical hypothesis testing method, introduced to the field of electrophysiology in the seminal paper by Eric Maris and Robert Oostenveld (Maris and Oostenveld, 2007), to circumvent the multiple comparison problems in the analysis of ERP's. Specifically, to test the cognitive processing effect on the attended speech events at a particular time *t*, the permutation test is performed in the following way:

1. Calculate the test statistic (mean value across epochs) of the epoched data for each experimental condition (attended events and unattended events).
2. Pool all epochs from both conditions.
3. Randomly divide the epochs into two subsets, with the number of epochs in each subset equal to the number of epochs in each experimental condition.
4. Calculate the test statistic (mean value across epochs) of each subset.
5. Repeat steps 3 and 4 a large number of times (1,000 times in this study) and construct the histogram of the test statistic.
6. The *p*-value of the test is the proportion of the number of random partitions that resulted in data at least as extreme as the observed data from step 1.

The cluster-based permutation test was used to determine the significance of the spatial and temporal locations of the effect, based on the ERP waveform and topography, respectively. The cluster-based permutation test follows a similar procedure to the permutation test described above. However, in the cluster-based permutation test, the test statistics are calculated at the cluster level. For instance, when testing the significance of the temporal location, the following steps are taken to find the test statistics for each permutation:

1. For each sample in the epoch, calculate the difference between the mean values of two experimental conditions (attended events and unattended events).
2. Find all clusters. A cluster is a set of temporally adjacent data points that have difference values larger (or absolutely larger for a two-sided test statistic) than a threshold.
3. Calculate test statistics for each cluster by summing the mean difference within a cluster.
4. The largest statistic among clusters is the permutation test statistic.

Note that, as highlighted in the original study (Maris and Oostenveld, 2007), the threshold used in step (2) to form clusters does not influence the false alarm rate of the test, but it does impact the test's sensitivity.

## 3 Results

Twenty-four subjects were included in the study and all subjects completed all four experiments. During the experiments, subjects were probed with a question after each trial, to allow the exclusion of subjects who were not following the attention instructions. However, all subjects had a satisfying rate of correct answers, therefore no subjects were excluded based on their behavioral responses. The individual accuracies for each paradigm are shown in Supplementary Table S1.

In the following sections, ERP quantification was performed by measuring local peaks of the waveform within the time window of significant duration found by the cluster permutation test.

### 3.1 Paradigm 1: word category oddball

**Figure 3:**
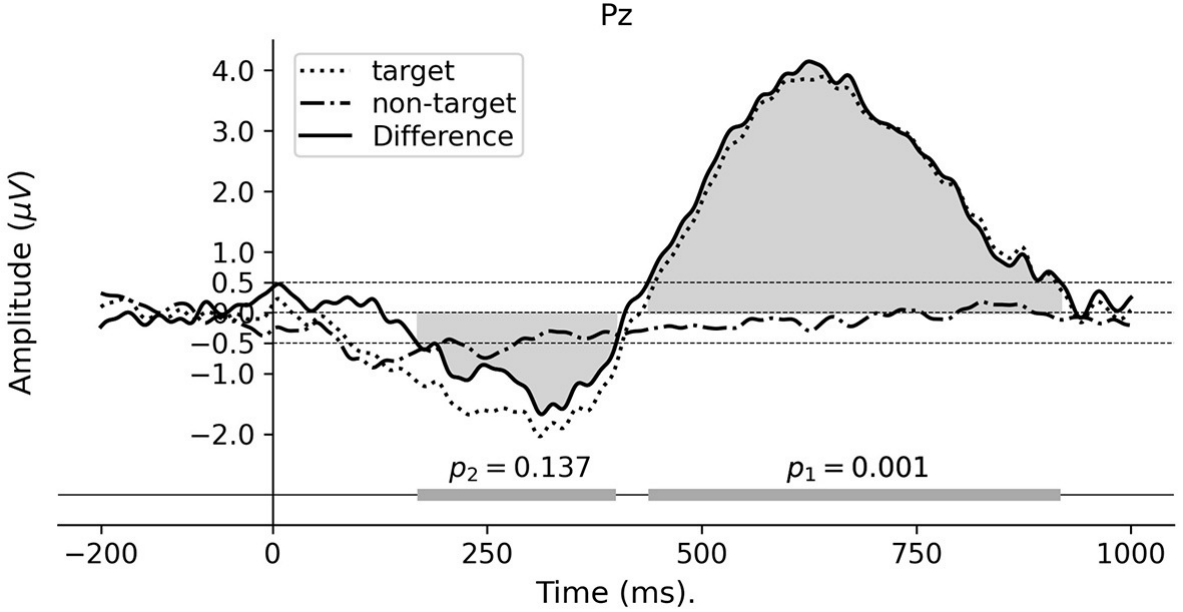
Grand average ERP waveforms for Paradigm 1. The ERPs are calculated from electrode Pz referenced to the average of all scalp electrodes. The horizontal bar at the bottom represents the detected temporal clusters and their corresponding p-values from the cluster permutation test for the difference ERP waveform.

The individual ERPs were calculated by averaging across epochs corresponding to the target and non-target words, using the analysis pipeline described in Section 2.5. The difference ERPs were obtained by subtracting the non-target ERPs from the target ERPs. As shown in Figure 3, the grand average difference ERP of electrode Pz demonstrates a positive going approximately from 400 to 950 ms and represents a significant difference between the two experimental conditions. To test the significance of the temporal location of the effect, a cluster permutation test was performed as described in Section 2.7. This threshold yielded two clusters in the difference waveform, see the bottom bar in Figure 3. The cluster permutation test resulted in a significant cluster within the time window 450–920 ms (*p* = 0.001), whereas the other cluster is insignificant (*p* = 0.137). The amplitude and latency of the ERP peak were measured at ~4.15 μV and 625 ms, respectively. This positive-going peak also appeared consistently within the significant cluster for all subjects (see Supplementary Figure S1).

**Figure 4:**
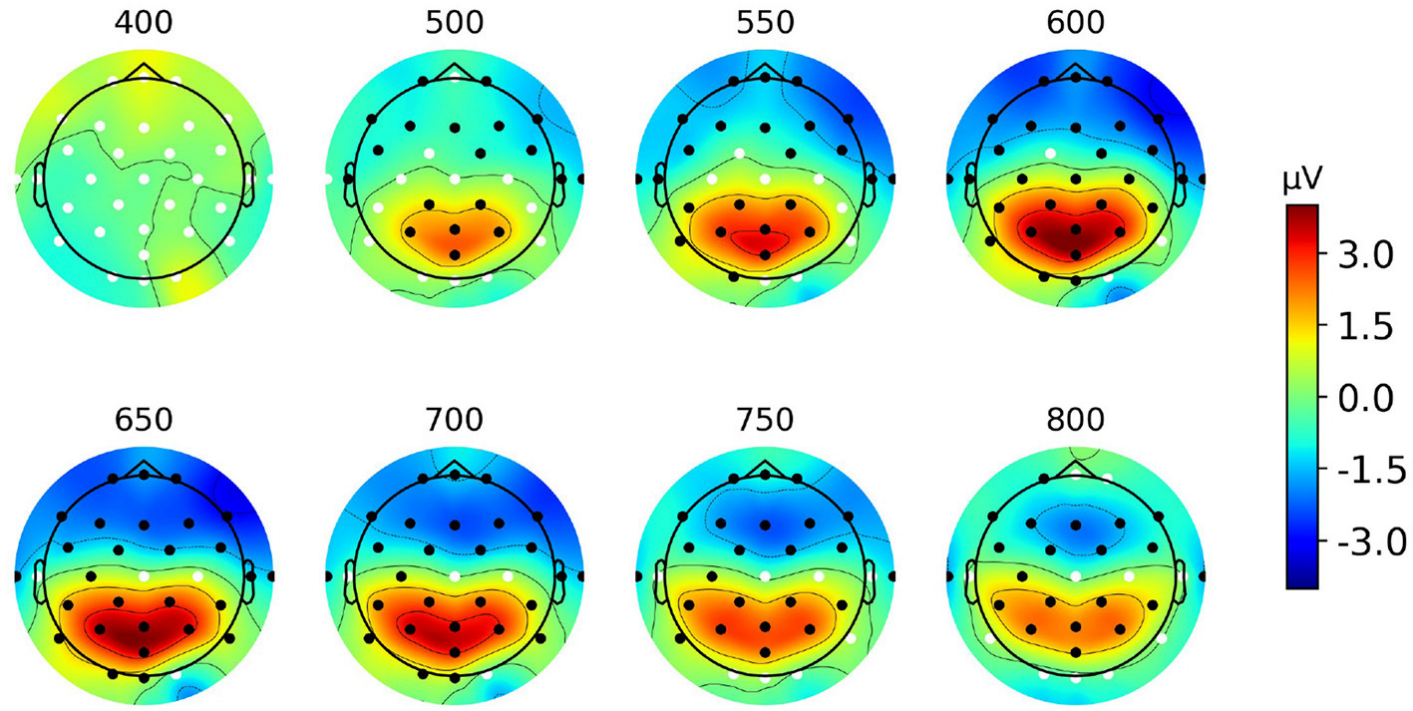
Grand average scalp topographies from 400 to 800 ms post-stimulus of the difference ERP for Paradigm 1. Black dots show significant electrodes and white dots show insignificant electrodes.

Figure 4 represents the scalp topographies of the grand average difference ERP. The areas marked by black dots indicate significant differences in effects between target and non-target ERPs as determined by the spatial cluster permutation test, using the same threshold from the temporal cluster test above. From Figure 4 we observe the emergence of a centro-parietal spatial cluster, starting around 500 ms and persisting until around 800 ms post-event (*p* < 0.001). Additionally, in each topography, there is another significant cluster at the frontal region which likely reflects the same neural source but with opposite polarity (conceptually representing the positive and negative ends of the source space dipole).

### 3.2 Paradigm 2 and 3: competing streams

This section presents the results from the analysis of the data from Paradigms 2 and 3, the competing streams paradigms, using the analysis pipeline described in Section 2.5.

**Figure 5:**
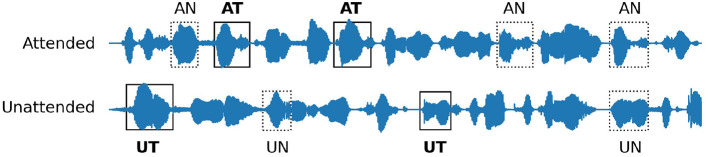
Definition of different experimental conditions. AT: target event in the attended stream, AN: non-target event in the attended stream, UT: target event in the unattended stream, UN: non-target event in the unattended stream. The target events (AT, UT) were predefined in each stream while the remaining words were considered as the non-target events (AN, UN).

**Figure 6:**
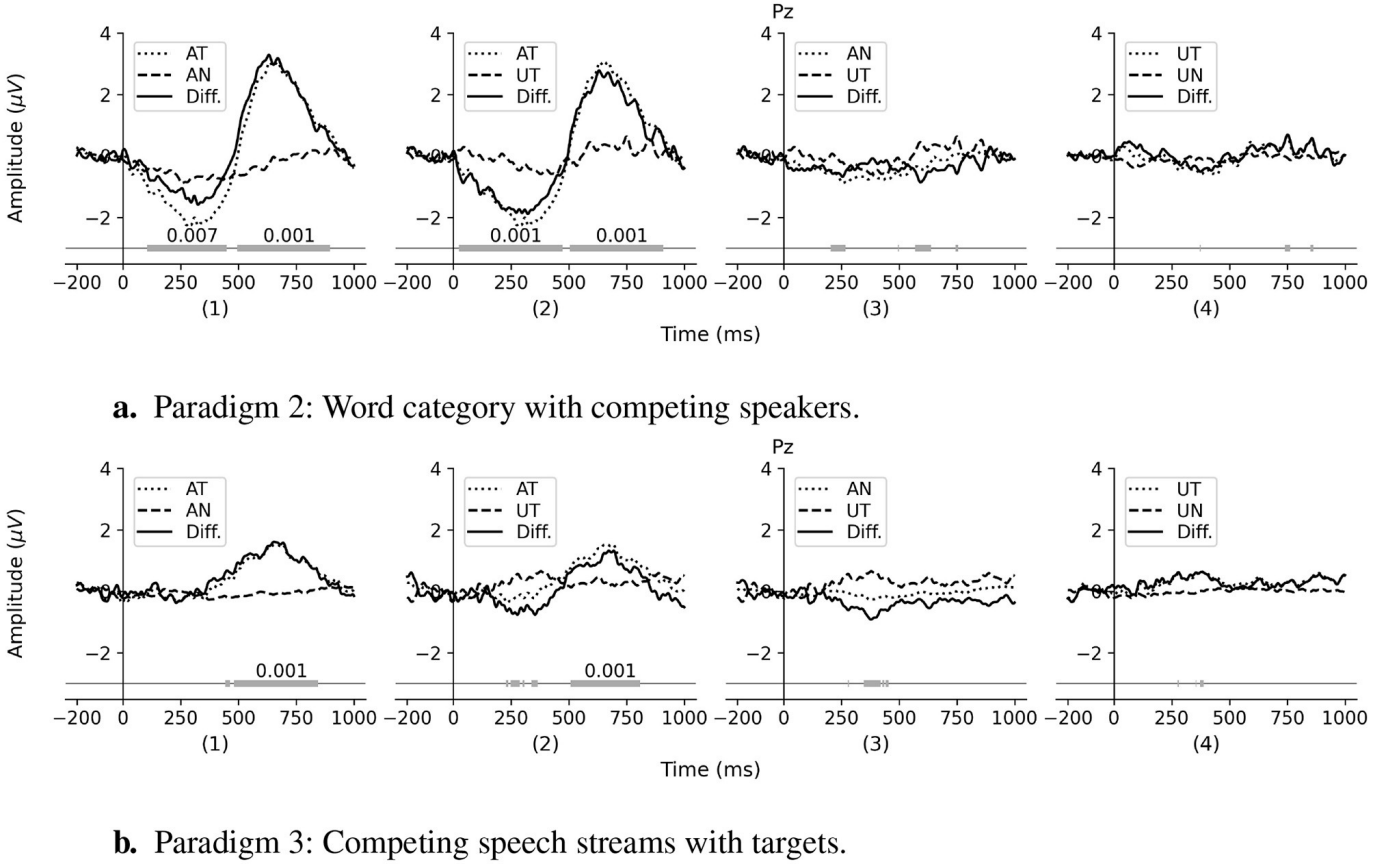
Comparison of the grand average ERPs of different experimental conditions from channel Pz in the multi-talker environment. **(A)** Results of Paradigm 2: Word category with competing speakers. **(B)** Results of Paradigm 3: Competing speech streams with targets. For each paradigm, panel **(1)**, **(2)**, **(3)**, and **(4)** represent the comparison of AT vs. AN, AT vs. UT, AN vs. UT, and UT vs. UN, respectively. The horizontal bars at the bottom of each panel represent the detected temporal clusters and their corresponding p-values (only for the significant clusters) from the cluster permutation test for the difference ERP waveforms.

For the presentation of the analysis results, we use the following terminology: AT (target event in the attended stream), AN (non-target event in the attended stream), UT (target event in the unattended stream), and UN (non-target event in the unattended stream). Figure 5 shows an example of a stimulus used in Paradigm 3, which comprises continuous speech streams. The target events (AT, UT) were predefined as the specific word classes in each story with the remaining words in each stream considered as the non-target events (AN, UN). In Paradigm 2, all events were discrete spoken words. For the sake of clarity, in the remainder of this paper, the term *AT waveform* refers to the waveform calculated by averaging all epochs of *AT events*. Similarly, the AN, UT, and UN waveforms refer to the average over the AN, UT, and UN epochs, respectively. For better comparison, Figure 6 shows the results of the analyses for both discrete and continuous speech paradigms.

#### 3.2.1 Paradigm 2: word category with competing speakers

In Paradigm 2, the two competing streams consisted of sequences of discrete spoken words. Four different analyses were performed according to four pairs of experimental conditions AT vs. AN, AT vs. UT, AN vs. UT, and UT vs. UN. The individual ERPs for each condition of each case were first calculated. The difference ERPs were obtained by subtracting the ERPs of the latter from the former in each pair. Finally, the grand average ERPs were calculated by averaging the individual ERPs. Figure 6A shows the grand average ERPs for four analyses. The bottom bars show the detected clusters and the p-values of significant clusters from the temporal cluster permutation test for the difference ERP waveforms.

Figure 6A.1 presents the results of the AT vs. AN comparison which compares the ERP waveforms of the target and non-target events in the attended stream. The target ERP (AT) has a clear positive deflection at around 632 ms with an amplitude of ~3.3 μV, whereas there are no noticeable deflections in the non-target ERP (AN). The cluster permutation analysis of the difference ERP reveals a significant cluster in the 500–900 ms time window (*p* = 0.001, α = 0.0125, Bonferroni corrected). Similarly, a comparison of the target ERPs in the attended stream (AT) and unattended stream (UT) are shown in Figure 6A.2, and the cluster permutation test reveals a significant difference in the time window 500–900 ms (*p* < 0.001, α = 0.0125, Bonferroni corrected). The difference ERP between AN and UT is shown in Figure 6A.3; the cluster permutation test did not find a significant difference (*p*>0.1 for all time points). Finally, the difference between UT and UN is shown in Figure 6A.4, and as for the AN vs. UT, there is no significant difference (*p*>0.1 for all time points).

**Figure 7:**
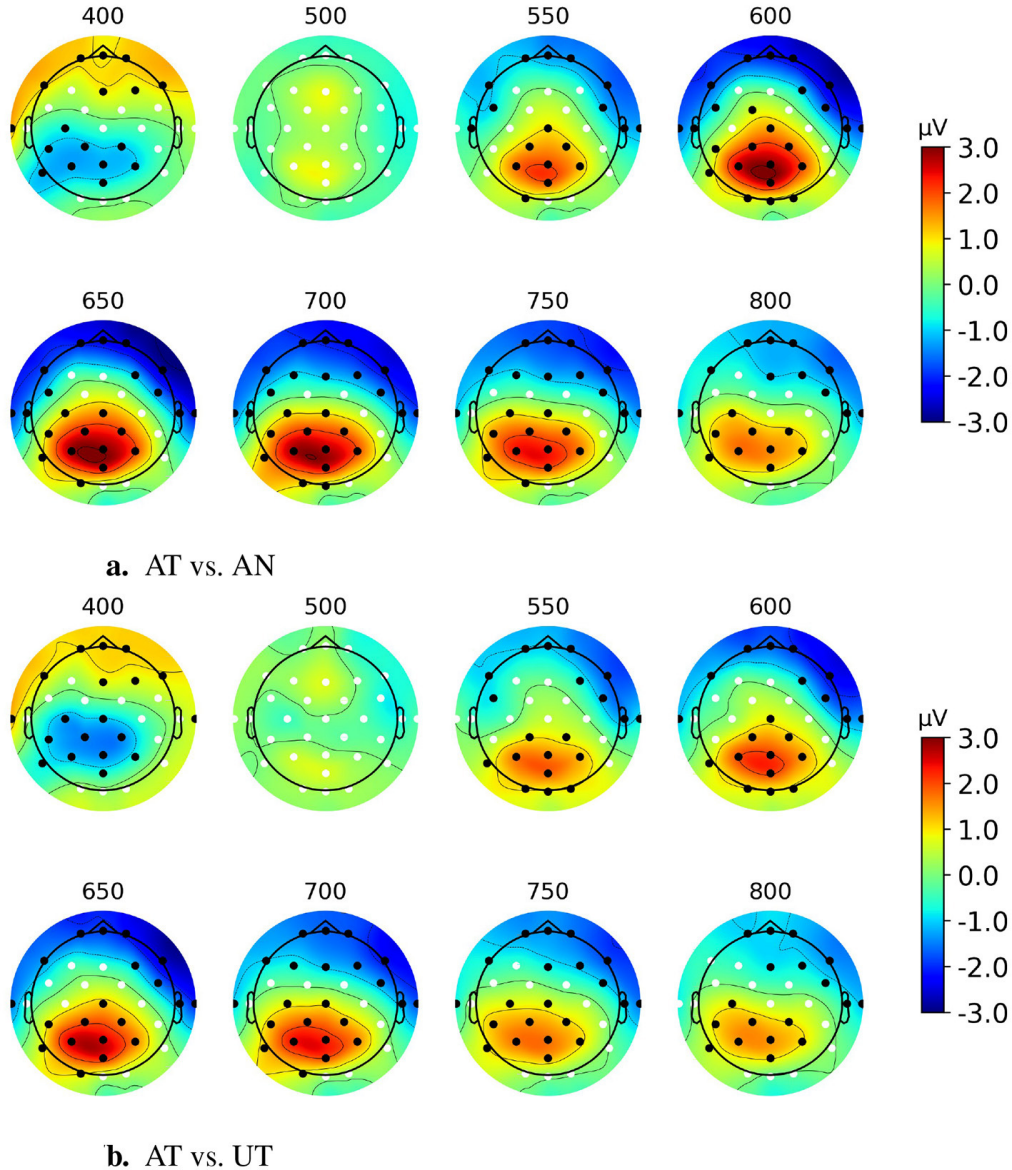
Scalp topographies of AT-AN and AT-UT difference ERPs between 400 and 800 ms post-stimulus for discrete event competing paradigm. Black dots show significant electrodes, and white dots show insignificant electrodes. **(A)** AT vs. AN. **(B)** AT vs. UT.

Figure 7 shows the scalp topographies, between 400 and 800 ms, of the AT-AN and AT-UT difference ERPs. The permutation test results show that the significant clusters (*p* < 0.01, α = 0.025, Bonferroni corrected for the black dots region) of a cognitive component generated by AT events in the competing stream paradigm are also at the parietal site. This is consistent with the observations from Paradigm 1 in Section 3.1.

#### 3.2.2 Paradigm 3: competing speech streams with targets

The data analysis followed the same procedures as used in Section 3.2.1, albeit with the modification that the AN and UN waveforms were computed from epochs containing all words except those selected as target events in the attended and unattended streams, respectively.

Figure 6B shows the grand average ERPs of four analyses for Paradigm 3. Similar to the results from the *word category with competing speakers* paradigm, the AT waveform is significantly different from the AN waveform (*p* = 0.001, α = 0.0125, Bonferroni corrected) and the UT waveform (*p* = 0.001, α = 0.0125, Bonferroni corrected) in the duration of 500–800 ms. However, the amplitude of the AT waveform is noticeably smaller (maximum amplitude 1.61 μV vs. 3.3 μV). The scalp topographies of two analyses: AT vs. AN and AT vs. UT show a similar pattern to that of the *word category with competing speakers* paradigm with the significant clusters at the parietal site (see Supplementary Figure S4). There are no noticeable differences between the three types of events: AN, UT, and UN.

To ensure that the grand average waveform represents a generalizable pattern across subjects, the individual difference ERP waveforms between AT and AN for both Paradigm 2 and Paradigm 3 are displayed in Supplementary Figures S2, S3, respectively. The individual ERP waveforms for Paradigm 2 show a consistent pattern across subjects. In contrast, the ERP waveforms for Paradigm 3 generally exhibit lower amplitudes, and a greater degree of variability between subjects, and appear noisier compared to those of Paradigms 1 and 2. Nevertheless, the grand average ERP is not driven by outliers or a small subset of subjects, as the majority the individual waveforms exhibit a very similar waveform as the grand average. These findings are consistent with the significant clusters observed for Paradigm 2 and Paradigm 3 in the 500–800 ms range.

### 3.3 Cognitive component in ear-EEG

**Figure 8:**
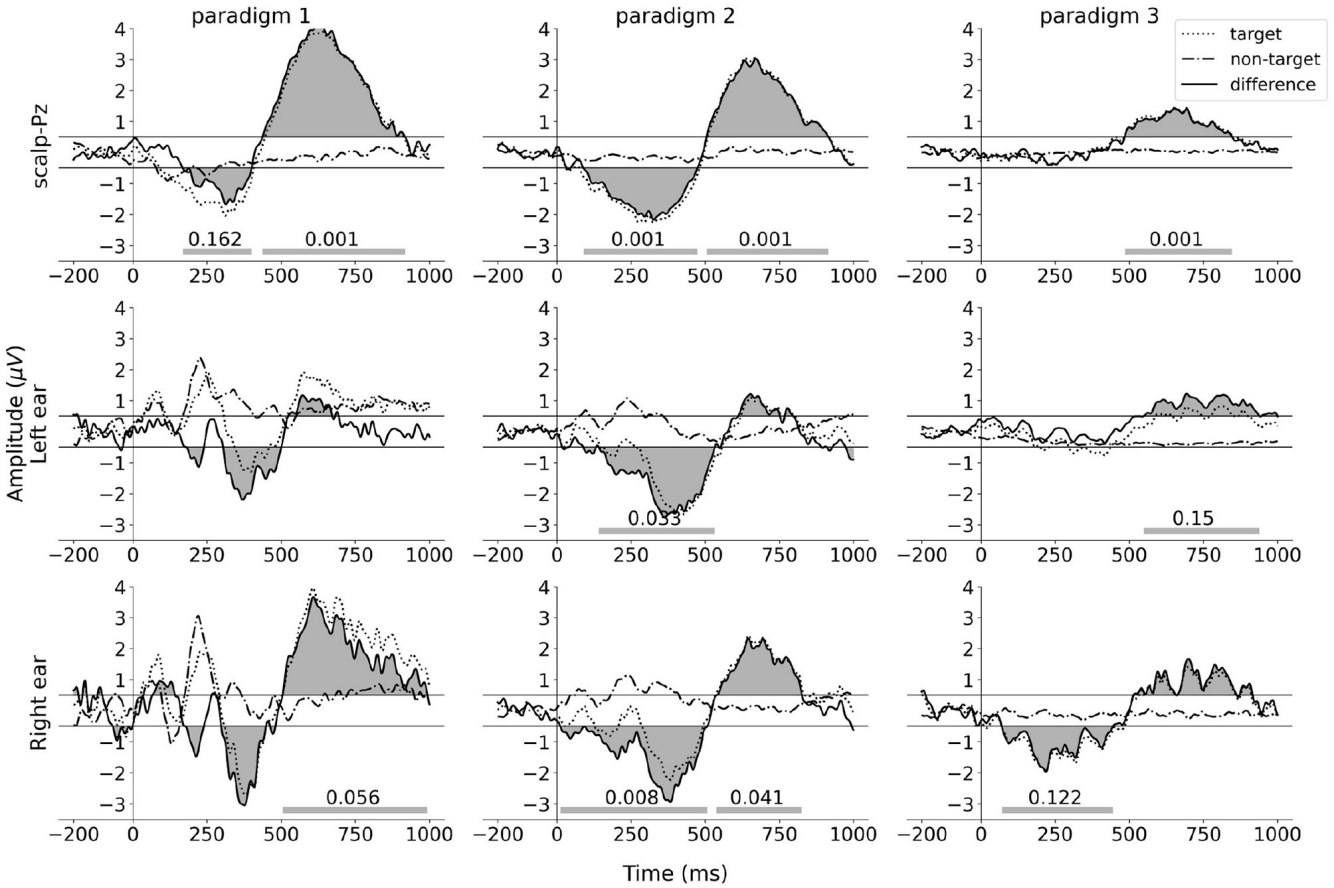
ERP and difference waves for scalp and ear-EEG signals. The dotted lines represent the target ERP waveforms. The dot-dash lines represent the non-target ERP waveforms. The solid lines represent the difference ERP waveforms. The top row presents the grand average ERP waveforms calculated from electrode Pz, with reference to the average of all scalp electrodes. The middle row and bottom row present the grand average ERP waveforms obtained from the output of the spatial filter applied to six left-ear electrodes, with reference to the average of these electrodes, and the output of the spatial filter applied to six right-ear electrodes, with reference to these electrodes, respectively. The gray areas between the difference ERP waveforms and the horizontal lines *y* = 0.5/*y* = −0.5 represent the clusters detected by the two-side permutation test with threshold 0.5. The cluster *p*-values lower than 0.2 are shown on the horizontal bars at the bottom of each subplot.

The ERP waveforms for the ear-EEG data were obtained using the spatial filtering method described in Section 2.6.2. Each column in Figure 8 shows the grand average ERP waveforms for each paradigm. The ERP waveforms for electrode Pz are presented in the top row to facilitate comparison. The filtered grand average ERP waveforms for the left ear and the right ear are displayed in the middle row and bottom row, respectively.

While the cross-task validation scheme did not find any significant cluster (*p*≥0.041, α = 0.025, Bonferroni corrected), the difference ERP waveforms of both ears exhibit a very similar pattern to the Pz channel ERPs across all three paradigms. To quantify the similarity, we calculated the cosine similarity between the scalp (Pz) and the ear ERP's, and the significance of the similarity was calculated from a permutation test distribution between the Pz and permuted target/non-target ear-ERPs. The result of this analysis is shown in Table 1. It is observed that in each paradigm, the similarity of ERP waveforms between the right ear and the Pz electrode is significant (*p* ≤ 0.022, α = 0.025, Bonferroni corrected). For the left ear, all similarity scores are >0.56 and are found to be less significant.

**Table 1:** Similarity scores and corresponding *p*-values between the ear and scalp ERPs for the three paradigms. All significant values (*p* < 0.025, Bonferroni corrected.) are in boldface.

**Ear**	**Paradigm 1**		**Paradigm 2**		**Paradigm 3**	
	**score**	*p* **-value**	**score**	*p* **-value**	**score**	*p* **-value**
Left	0.563	0.140	0.715	0.050	0.734	0.050
Right	0.802	**0.017**	0.910	**0.001**	0.787	**0.022**

## 4 Discussion

### 4.1 Paradigm 1: word category oddball

The word category oddball paradigm, using discrete spoken words as targets and non-targets, showed a significant difference wave from the Pz electrode in the 450–900 ms interval, with a peak amplitude around 4.15 μV at ~625 ms post-event. Although the peak appears with a significantly longer latency than reported in other studies: 240–350 ms (Squires et al., 1975) and 200–500 ms (Polich, 2007), due to the similarity in the experimental paradigm and the cognitive task evoking the response, we conjecture that the response and underlying mechanism are the same as for the P3b component. The longer peak latency may be explained by higher stimulus evaluation timing due to the use of spoken word stimuli, task processing demand, and higher task complexity, which are aligned with the findings in other P300 studies (Polich, 2007; Kutas et al., 1977). In this study, the task includes the semantic parsing of the meaning of the events and classifying them into two categories which are similar to the visual-presented word class categorization tasks with the reported latency of up to 780 ms depending on the reaction time (Kutas et al., 1977). In another study (Leckey and Federmeier, 2020), it is argued that P600 component is a representation of P3b where the semantic/syntactic mismatches act as oddball events. This further substantiates that the ERP identified in this study is related to a long-latency P3b component. Moreover, a sequence of scalp topographies from 400 to 800 ms shown in Figure 4 demonstrates that the positive deflection has a maximum in the central parietal region, which further supports and substantiates that the deflection reflects a P3b component.

### 4.2 Paradigm 2 and 3: competing streams

This section discusses how the studied component is impacted by both the multi-talker environment and normal speech context.

#### 4.2.1 Paradigm 2: word category with competing speakers

The results shown in Figure 6A illustrate how the studied component is affected by the attention in the multi-talker environment. The positive deflection of the difference ERP waveform of the AT and AN events is shown in Figure 6A.1 suggests that the studied component can also be elicited by the *word category oddball* in the presence of competing speakers. The unattended stream in this case plays a role as a distractor, i.e., background noise. The significant difference between AT and UT in the duration 500–900 ms (*p* = 0.001), see Figure 6A.2, suggests that the studied component is specifically elicited by the attended oddballs, rather than by the mere presence of a semantic oddball. In other words, although semantic oddballs are present in both the attended and unattended streams, the cognitive component is only evoked by oddballs within the attended stream. This is a remarkable characteristic making it a promising component for addressing the AAD problem. Moreover, we did not find any significant differences between the AN and the UT, which indicates that the target events in the unattended stream do not attract more attention than the non-target events in the attended stream. The result from Figure 6A.4 demonstrates that when the subject ignores the sound source, all the events in that source do not generate any difference in ERP waveforms and are almost equally unattended. This again clarifies the aforementioned hypothesis that the selective attention mechanism plays an important role in triggering and manipulating the P3b component rather than the presence of a semantic oddball itself. The results from this paradigm demonstrate that, in a multi-talker environment, the cognitive ERP component can be elicited and observed at the parietal site only if the oddball events appear in the attended stream.

#### 4.2.2 Paradigm 3: competing speech streams with targets

The attended target (AT) ERP and the difference wave (AT minus AN) elicited by Paradigm 2 shown in Figure 6A has the same morphology as the target ERP and difference wave (target minus non-target) elicited by Paradigm 1 shown in Figure 3. This suggests that the responses are related to the same underlying neural source in both the single speaker and the competing speaker paradigm.

However, the stimuli, which were sequences of discrete speech events, were still rather artificial and did not resemble real-life speech signals. Therefore, the results from Paradigm 2 in Section 3.2.1 are not sufficient to ensure that these responses will be present, let alone detectable, in more naturally occurring stimuli. To address this, in Section 3.2.2, we presented results on how the cognitive component manifests in a “more realistic” listening situation using natural, continuous speech stimuli.

The results in Section 3.2.1 show that identical component can be observed in a multi-talker environment. However, the stimuli, sequences of discrete speech events, were quite simple and well-formatted, creating an unrealistic listening context. Therefore, it is premature to conclude that the studied cognitive component is a solid and promising candidate to apply in any speech-based BCI application. In Figure 6, all the ERP waveform patterns in the continuous speech paradigm (bottom row) are case-wise similar to those in the discrete speech paradigm (top row). However, the peak amplitude of the AT waveform in the latter is noticeably smaller. It is reasonable because of the less distinct nature of the target events in the more realistic listening scenario, i.e., the less salience of the events which mainly modulates the amplitude of the P3b component. Additionally, in the context of listening to a story, subjects tend to be less attended to the target events due to lack of time. The topographies again confirm that the neural source location when we use the natural speech stimuli is also around the parietal site which is identical to the location of the studied component in the single stream paradigm (Figure 4) and in the discrete event competing streams (Figure 7). We observe that the topographies of Paradigms 2 and 3 exhibit a slight left lateralization compared to those of Paradigm 1. Numerous previous studies, including Wernicke's foundational work on the anatomical basis of aphasia (Wernicke, 1969), have established that language processing is largely left-lateralized (Hickok and Poeppel, 2007). Of particular relevance to the current study is the significance of Wernicke's area and its role in language comprehension, which is located near the C3, P3, and P7 electrodes. Thus, a plausible explanation for the observed left lateralization is that the tasks in Paradigms 1 through 3 progressively increase in demands related to language comprehension.

The results from this paradigm suggest that the studied component is observable even in a realistic listening scenario where the subject attends to one speech stream among multiple streams. The latency and spatial distribution of the component appear to be independent of how closely the stimulus resembles speech in realistic situations. Also, the level of attention significantly affects the amplitude of this component. All else being equal, the more attention allocated to an event, the larger the amplitude of the component. It is obvious that the target words in the attended streams, as defined by the task in the experiment, become important words in the speech context. In a specific speech context, it is very likely that the important words catch the most attention and evoke a larger P3b component. Although determining the important words in real-time speech context is a challenging task, with the help of advanced semantic and syntactic analysis in language models, this component can be further utilized to solve the AAD problem where predefined target words are not available. Alternatively, training temporal response function (TRF) to predict the brain response to speech signals may help identify which parts of different speech complexities, including long phrases or sentences, cause stronger responses. Based on that a further analysis of the cognitive response to these parts can be conducted.

In summary, attention to specific events in competing speaker paradigms, whether involving discrete words or continuous speech streams, evokes a cognitive component. This component is particularly observable in the parietal region. In the competing speech streams paradigm, which is a more realistic listening situation, the amplitude of the component seems to be smaller.

### 4.3 Studied component in the ear-EEG signal

Despite the inherent challenges of high inter-subject variance and low SNR in the in-ear EEG data, the application of spatial filtering yielded similar in-ear ERPs to those observed on the scalp for all three paradigms (all similarity scores are >0.56 for both ears). This demonstrates that the potential of the studied component, elicited by speech events, is observable not only at scalp locations but also at both ears and can be measured using in-ear electrodes and decoded by spatial filters. This opens up possibilities of utilizing this component with a compact and miniaturized in-ear EEG setup to address the AAD problem and other BCI applications.

Results from the statistical test show that the studied component is significant in training tasks but not in validation tasks. This observation suggests a potential issue of overfitting the spatial filters due to the limited amount of training data. We believe that this problem could be mitigated by increasing the volume of training data. Furthermore, the low SNR characteristic of ear-EEG could present a barrier to achieving significant results. This reveals challenges for future research to explore more advanced signal processing methods to utilize the studied component in the in-ear EEG signals.

## 5 Conclusion

Through the utilization of natural speech stimuli and cognitive tasks, a study of cognitive components related to auditory attention was conducted on both scalp and in-ear EEG devices. The findings demonstrate that the cognitive processing of natural speech events can be observed at parietal electrode sites and typically peaks at ~625 ms, which is more likely to be a P3b component. Furthermore, we have also shown that the studied component is significantly observable in the attended speech stream of a multi-talker environment and its amplitude is influenced by the level of attention given by the brain to the speech events. These results suggest that the studied component carries information that is useful for decoding auditory attention. Additionally, spatial filtering played a pivotal role in extracting the cognitive component from the ear-EEG signals, thus marking a significant advancement toward cognitively controlled hearing devices.

## Data Availability

The datasets presented in this article are not readily available due to ongoing data analysis. Requests to access the datasets should be directed to the corresponding author.

## Supplementary material

Supplementary material Cognitive component of auditory attention to natural speech events.
