# Supplementary material for: Cognitive component of auditory attention to natural speech events
Source: Front Hum Neurosci. 2025 Jan 6;18:1460139. doi: 10.3389/fnhum.2024.1460139 (PMC11743694; doi:10.3389/fnhum.2024.1460139)
Supplement: Supplementary material — Cognitive component of auditory attention to natural speech events. [file Data_Sheet_1.pdf]

Supplementary Material

1 SUPPLEMENTARY FIGURES

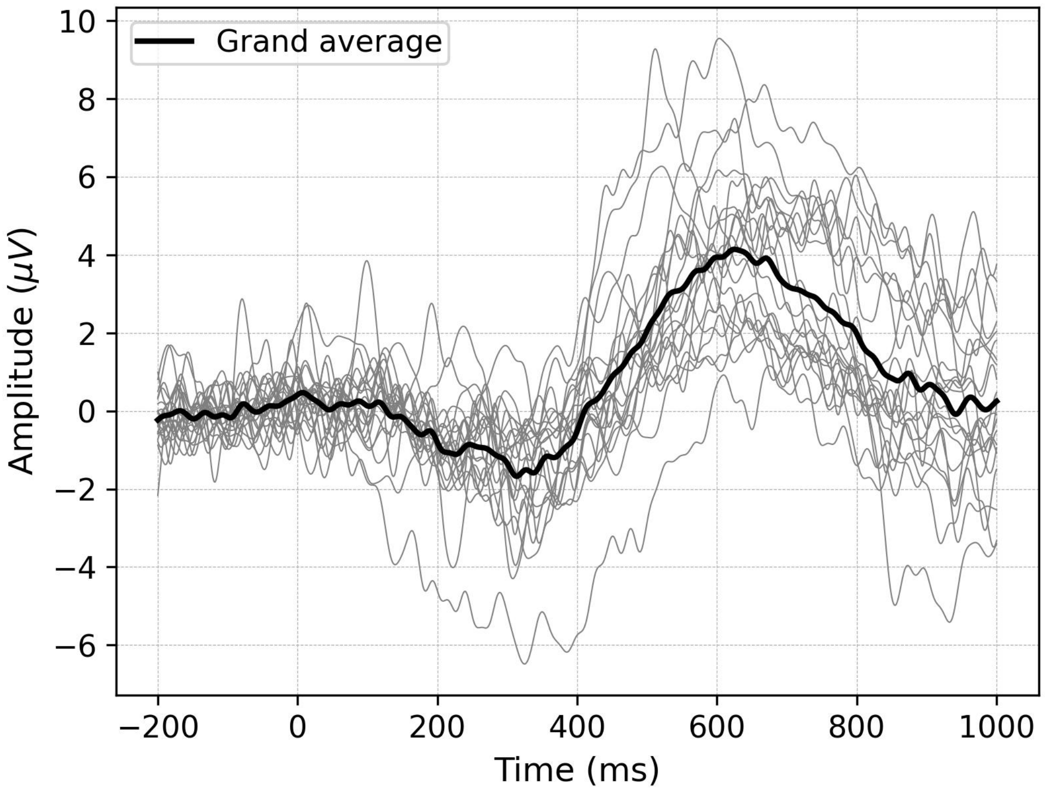

Figure S1. Individual average and grand average ERP waveforms for Paradigm 1: *word category oddball* paradigm. The ERPs are calculated from electrode Pz referenced to the average of all scalp electrodes.

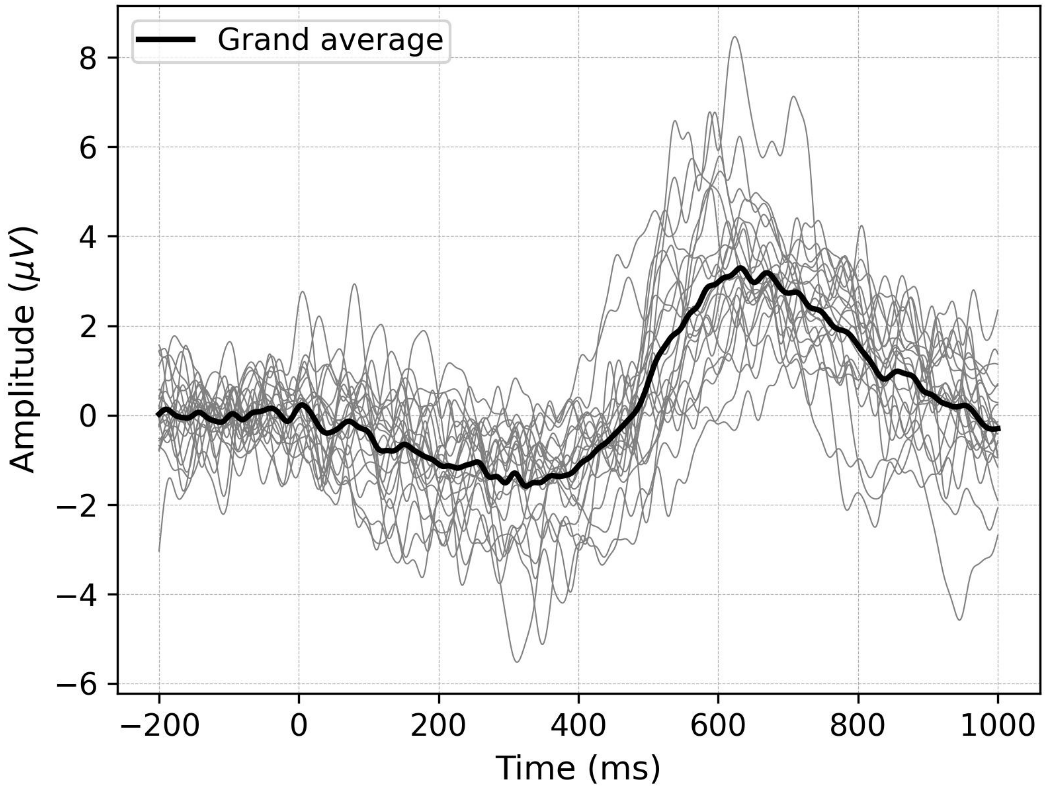

Figure S2. Individual average and grand average ERP waveforms for Paradigm 2: *Word category with competing speakers*. The ERPs are calculated from electrode Pz referenced to the average of all scalp electrodes.

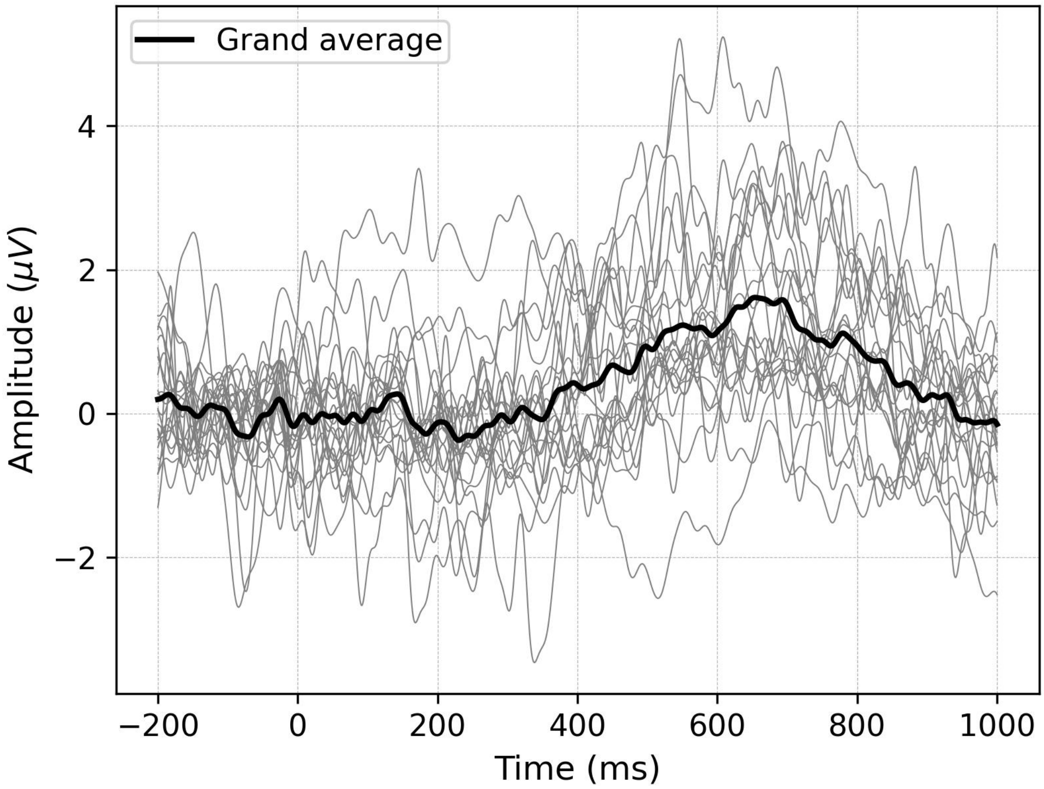

Figure S3. Individual average and grand average ERP waveforms for Paradigm 3: *Competing speech streams with targets*. The ERPs are calculated from electrode Pz referenced to the average of all scalp electrodes.

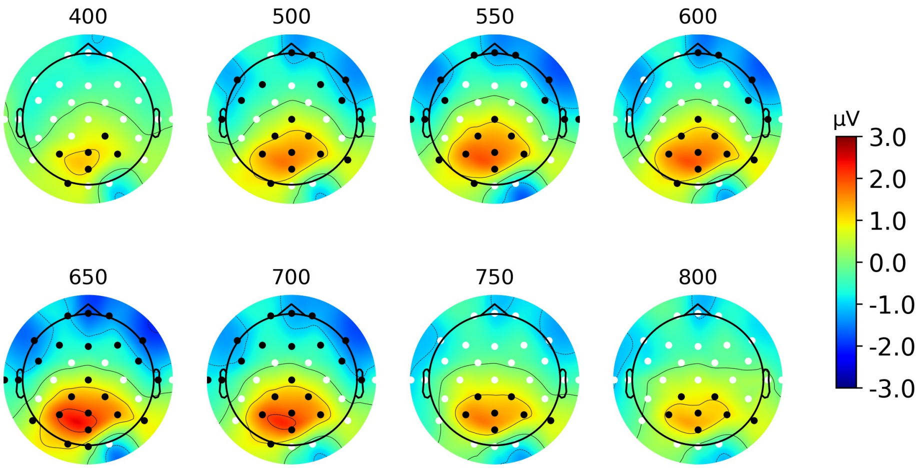

Figure 4a. AT vs. AN

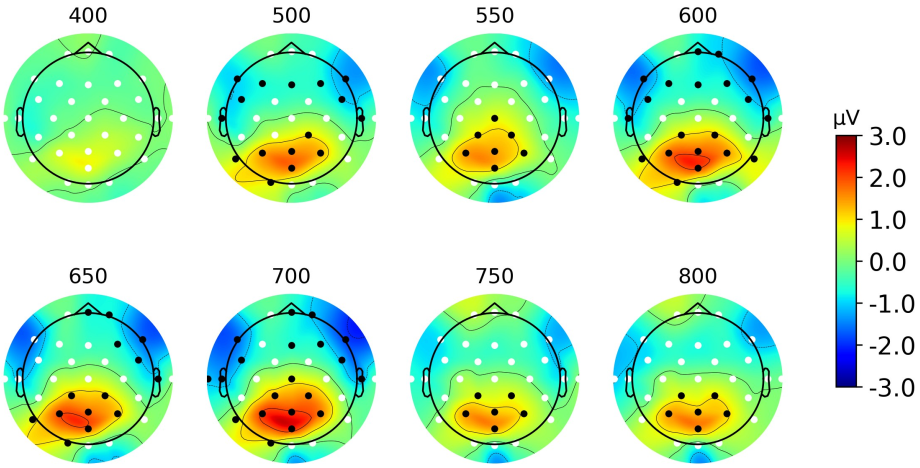

Figure 4b. AT vs. UT

Figure 4. Scalp topographies of AT-AN and AT-UT difference ERPs between 400 and 800 ms post-stimulus for competing speech streams paradigm. Black dots show significant electrodes, and white dots show insignificant electrodes.

2 SUPPLEMENTARY TABLES

Table S1. Subject response accuracies(%) for three paradigms.

Paradigms	S1	S2	S3	S4	S5	S6	S7	S8	S9	S10	S11	S12
1	93.8	93.8	100	93.8	100	87.5	100	87.5	100	100	87.5	100
2	90	85	95	100	95	95	100	80	90	100	85	95
3	75	70	80	70	65	70	70	60	80	85	80	85
**Paradigms**	**S13**	**S14**	**S15**	**S16**	**S17**	**S18**	**S19**	**S20**	**S21**	**S22**	**S23**	**S24**
1	100	87.5	93.8	100	93.8	100	87.5	100	93.8	100	100	87.5
2	100	90	100	90	95	95	95	95	95	95	95	90
3	80	65	75	75	80	70	90	60	65	80	80	70
